# Too Lucky to Be a Victim? An Exploratory Study of Online Harassment and Hate Messages Faced by Social Media Influencers

**DOI:** 10.1007/s10610-023-09542-0

**Published:** 2023-06-07

**Authors:** Noelia Valenzuela-García, Diego J. Maldonado-Guzmán, Andrea García-Pérez, Cristina Del-Real

**Affiliations:** 1 Department of International Public, Criminal and Procedural Law, University of Cadiz, Av. De La Universidad S/N, 11405 Jerez de La Frontera, Cádiz, Spain; 2 Institute of Security and Global Affairs, Leiden University, The Hague, The Netherlands

**Keywords:** Celebrities, Cyber offences, Cyber-enabled crime, Stalking, Netnography

## Abstract

Influencers are persistently exposed through social media. Once almost unapproachable, celebrities are now open to daily interaction with the public. From comments, polls, emails, and even private messages, the public can engage with their celebrities with a mere click. While this engagement provides influencers with advantages, it also renders them particularly susceptible to online harassment and toxic critics. This paper investigates the characteristics, impact, and reactions to cyber victimisation among social media influencers. To accomplish this objective, the paper presents the findings of two studies: a self-reported online victimisation survey conducted among Spanish influencers and an online ethnography. The results indicate that over 70% of influencers have encountered some form of online harassment and toxic critics. Cyber victimisation, its effects, and reactions vary across socio-demographic characteristics and the influencers’ profiles. Furthermore, the qualitative analysis of the online ethnography reveals that harassed influencers can be classified as non-ideal victims. The implications of these findings for the literature are discussed.

## Introduction

On 25th January 2018, Spanish influencer Alexandra Pereira, popularly known as "*Lovely Pepa*",^1^ caused a stir on social media with a 30-min video where she denounced the persistent harassment she had been subjected to for several years (Pereira, 2018). According to the influencer, she had received over 70,000 hate messages on the Vogue forums^2^ alone in the last eight years. Her video went viral, leading to the cancellation of the popular Vogue magazine forum (El País, 2018a). In the video, the influencer, who has over 1.5 million followers, highlighted that she was not the only victim of this menace. The normalisation of online harassment against influencers on social media is increasingly evident. Another recent example involved the renowned Spanish influencer Aida Domenech, known as "*Dulceida*", who has 3.1 million followers on Instagram. During a segment on "*El Hormiguero*,"^3^ she confessed about the severe harassment she endures daily. Some of the comments were so severe, like "*if I see you with an LGBT flag again, I’ll burn you alive*," that she had to report them to the police (Robles, 2022). These are not isolated cases as there are numerous daily examples of harassment against influencers on the web, with recent studies demonstrating increased awareness of this problem among the younger generation (Martínez Valeiro & Mayagoitia Soria, 2021).

The internet, especially social media, is a fertile environment for promoting hatred, harassment, and noxious communication. According to the most recent survey conducted by Pew Research Center in 2020, with 10,093 adult participants from the United States, 41% of them reported encountering some form of online harassment (Vogels, 2021). Existing literature on online harassment has examined its association with gender (e.g., McLean & Griffiths, 2019; Nadim & Fladmoe, 2021), the perceived severity of the issue among law enforcement officials (Holt et al., 2019), the socio-demographic and psychological factors that impact the perpetration of harassment, the experiences of victims and their reactions (e.g Celuch et al., 2022; Choi et al., 2022; Moneva et al., 2020), and the distinct victimisation faced by professionals, including scholars (e.g., Veletsianos et al., 2018) and journalists (e.g., Chen et al., 2020; Waisbord, 2020). Although the profiles of journalists and influencers may share similarities, with both requiring a significant online audience engagement, studies focusing specifically on social media influencers are scarce.

To date, only two studies have addressed the issue of online harassment among influencers to the best of our knowledge, both of which originate from the field of communication studies. The first study, conducted by Martínez Valeiro and Mayagoitia Soria (2021), examined hate speech, harassment, and self-censorship among social media influencers in Spain. The authors collected data from a seven-month online participant observation of the top ten Spanish influencer accounts with the most followers, along with focus groups and a survey of university students. The study discovered that influencers opt not to share publicly the hate messages they receive, frequently leave the platforms where they feel the most harassed, and self-censor their content to avoid becoming victims. While younger audiences recognise the existence of such harassment, they see it as a common feature of social media (Martínez Valeiro & Mayagoitia Soria, 2021, p. 30). The second study, conducted by Duffy et al. (2022), explored the perpetrators of harassment by analysing 400 comments posted by a female-dominated community of anti-fans in the well-known influencer hate blog, Get Off My Internets (GOMIBLOG). According to this study, critiques of influencers may be associated with a profound frustration with influencers who profit from representations of femininity deemed "unrealistic" (Duffy et al., 2022, p. 1670), which explains why the majority of harassment against women influencers is committed by female anti-fans – a type of *digital horizontal violence*.^4^

Despite the valuable insights provided by the two aforementioned studies, an examination of this issue through criminological and victimological perspectives is still lacking. Understanding the phenomenon of influencer harassment on social media is critical not only for effectively addressing this type of victimisation but also because individuals who are frequently exposed to this form of victimisation may become desensitised to violence, toxic communication, and hate speech on the internet (Soral et al., 2020). This study presents the outcomes of two preliminary investigations on the topic of online harassment of influencers in Spain. The first study employs primary data from a survey of 76 Spanish influencers, while the second study is a virtual ethnography of the popular social network Instagram. Through these two studies, we explore various aspects of this problem, including the nature of victimisation, the profile of the perpetrators, the profile of the influencer victims, the rationale behind the abuse, and its consequences. This study adopts a mixed research approach as it recognises that influencer harassment, being a novel topic, is best explored through a combination of quantitative and qualitative methods to gain a comprehensive understanding of the issue. This approach is recommended when a single source of evidence is insufficient to comprehend a research phenomenon (Creswell & Plano-Clark, 2007). The use of mixed methods compensates for the weaknesses of each approach (Onwuegbuzie & Leech, 2005). In this study, the survey provides direct experiences of influencers who have been victims of harassment, complementing the data from virtual ethnography that may not sufficiently explore victims’ experiences. Moreover, the virtual ethnography results supplement the survey’s sample size.

The paper is structured as follows. Section 2 presents the literature review, where we introduce the concepts of social media influencers and online harassment and discuss influencers as non-ideal victims. Section 3 describes the objectives of the two studies. The first study is presented in Sect. 4, including the methods and the result. The same structure is followed for the second study, described in Sect. 5. The discussion of the two studies is presented together in Sect. 6. Finally, Sect. 7 summarizes the contribution of this paper and proposes future research avenues.

## Literature Review

### Social Media Influencers

The origin of influencers can be traced back to the rise in popularity of the first *Camgirls* in the 1990s, who pioneered video blogs featuring live or recorded images and online texts, such as reviews or diaries (Senft, 2008). Building on this research, Senft developed the concept of the *microcelebrity* to describe individuals who sought fame through new technologies and established an online persona as a personal brand (Senft, 2013). This earlier concept shares several characteristics with today’s influencers, who are ordinary individuals that have attained celebrity status and the privileges associated with fame (Turner, 2006) through the celebrity economy fostered by cyberspace (Abidin, 2018). The popularisation of modern social networks has since established ‘*influencing*’ as a professional career with its own ecosystem and economics (Abidin, 2017). Influencers draw on different types of economic, cultural, technological, or social capital to pique the audience’s interest due to their uniqueness, exclusivity, rarity, or everyday nature (Abidin, 2018). This way, influencers develop strong bonds of trust with their followers, eventually emerging as opinion leaders capable of significantly influencing their followers’ decision-making (Arora et al., 2019).

The literature identifies five types of influencers, depending on the platform they primarily use to share their content. These include the *blogger*, who periodically publishes online texts on any subject matter, often on Twitter; the *youtuber* or *vlogger*, who communicates through recorded and extensive videos; the *instagramer*, who shares images and short videos on Instagram; and the two categories that have seen the most growth during the Covid-19 pandemic, the *streamer*, who typically interacts with the public through video broadcasts and live chat rooms, representing gamers, and the *tiktoker*, whose digital content consists of short, live videos via TikTok (Kádeková & Holienčinová, 2018). Khamis et al. (2017) and Schouten et al. (2020) posit that there is also a distinction between ‘traditional’ celebrities, such as actors, singers, supermodels, TV show presenters, or athletes who acquired their fame thanks to their professional talent, and social media influencers, who gained their status due to their expertise with social media platforms. However, it is important to note that there is no clear separation between traditional celebrities and influencers, as the latter can become famous people because they have been able to leverage their expertise with social media platforms to showcase their talent.

### Online Harassment and Hate Messages

Online harassment refers to the deliberate behaviours that intend to cause harm or discomfort to one or more users (Smith et al., 2008). Some scholars have identified its repetitive and prolonged nature, as well as the negative psychological, material, or social consequences it has on the victim (Bégin, 2018; Patchin & Hinduja, 2006). In this article, we adopt a broad definition of online harassment as any intentional behaviour directed towards influencers, irrespective of whether the perpetrator performs the behaviour once or repeatedly. We contend that the accumulation of such behaviours can amount to the continuity required to characterise harassment. For instance, if multiple individuals contact the influencer with similar abusive and hateful messages, the effect of such messages could be similar to the impact of repeated contacts by a single harasser. Although legally, each individual’s action may not attract punishment, criminologically, the cumulative impact can be just as harmful. Thus, we accept a concept of online harassment that encompasses various behaviours, such as threats, false accusations, identity theft, data or equipment damage, computer monitoring, among others, as proposed by Bocij and McFarlane (2002).

The scientific literature has approached the topic of online harassment from various angles. For instance, researchers have examined the harassment of different age groups, such as adolescents (Lwin et al., 2012) and adults (Ronzón-Tirado et al., 2022); and specific professional groups, such as journalists (Chen et al., 2020; Kantola & Harju, 2021), *youtubers* (Salian & Ghosh, 2022), and *gamers* (Jagayat & Choma, 2021). Online harassment against journalists affects both genders, but women appear to face greater toxicity, which can be attributed to their gender or sexuality (Chen et al., 2020; Holton et al., 2021; Kantola & Harju, 2021). Consequently, many journalists are contemplating abandoning social media platforms due to the lack of effective protection measures (Holton et al., 2021; Kantola & Harju, 2021).

The scientific literature has associated online harassment with Cohen and Felson’s (1979) routine activities theory (RAT). Research has indicated that RAT can be employed to develop strategies that aim to prevent bullying in online environments (Lindgren, 2018; Morillo Puente & Ríos Hernández, 2022; Vakhitova et al., 2019). Factors such as frequent use of social media, a large number of followers, having numerous strangers on friends lists, and frequent posting and updating were identified as predictors of online bullying (Näsi et al., 2017; Reyns et al., 2011). Although the applicability of RAT in the context of public figures and celebrities has been studied less frequently, Kumar et al. (2021) analysed harassment against female influencers on Twitter and found that those who expressed their opinions honestly and openly experienced greater levels of harassment. Many of these influencers eventually opted to restrict their online activities, which would not be a practical solution, given that their livelihood depends on continuously producing online content.

The scientific literature has attempted to elucidate the factors that drive an offender to engage in online harassment via social networks. There are three main factors that contribute to this phenomenon. Firstly, the concept of *online disinhibition* (Cheung et al., 2020) arises from the anonymity and identity protection provided by online platforms. This leads harassers to behave without concern for the consequences of their actions (Wright et al., 2019), engaging in behaviours that they would not exhibit in the physical world (Lowry et al., 2016; Sanfilippo et al., 2017; Wong et al., 2018; Wright et al., 2019). This sense of impunity and disinhibition may be a potential cause of harassment directed at online influencers. The second factor relates to *inequity aversion* (Fehr & Schmidt, 1999), whereby the harassment of influencers may be motivated by the perception of inequality in terms of the luxurious lifestyle they portray on their social media accounts. Finally, the concept of *fair punishment* (Dagger, 1993) is also relevant. In a society governed by a set of behavioural norms, punishment is imposed when an individual acts outside these boundaries. In the case of online harassment, it can be viewed as a form of social punishment meted out against individuals who have acted in a socially deviant or reprehensible manner. For instance, a study found that online harassment is perceived as more deserved and justifiable, albeit not more appropriate, when directed at an individual who has committed a crime (Blackwell et al., 2018). The same mechanism may ‘justify’ harassment directed at influencers who are seen to have violated social norms (e.g., by speeding, not wearing a seatbelt, or evading taxes).

### The (Non-)Ideal Victim

In 1986, Nils Christie posited that the designation of ‘victim’ not only hinges on having undergone an objective process of victimisation, but also on the recognition of it by society. Such recognition is brought about by multifaceted processes of cultural, social and symbolic significance, that vary across societies and eras (Holstein & Miller, 1990). The ‘ideal victim’ refers to ‘a person or a category of individuals who – when hit by crime – most readily are given the complete and legitimate status of being a victim’ (Christie, 1986, p. 18). Christie identifies six attributes of an ideal victim: she or he (i) is vulnerable and weak (e.g., female, elderly), (ii) is involved in a noble task, (iii) cannot be blamed, (iv) was harmed by malign forces or actors, (v) that cannot be specifically identified, and (vi) the victim’s status does not conflict with other interests. Influencers may lack some of these attributes, which could explain why they are not viewed as ideal victims and why this denial of their status justifies the violent behaviour directed at them.

The first attribute of the ideal victim is weakness, which makes them appear harmless and evokes empathy and sympathy. The Content Model stereotype later expanded on this attribute (Fiske et al., 2002). According to the authors, stereotypes are formed based on two primary dimensions – warmth and competence – and their interaction results in four distinct emotions: paternalistic (high warmth, low competence), admiration (high warmth, high competence), contemptuous (low warmth, low competence), and envious (low warmth, high competence). The ideal victim would elicit feelings of paternalism or even admiration (Bosma et al., 2018). Therefore, an influencer who evokes feelings of envy or contempt may not be perceived as weak, making it harder to establish and maintain their victim status. However, influencers are likely to evoke these emotions as they lead a luxurious lifestyle that is exposed to public scrutiny.

Influencers may lack other attributes that are necessary to be considered ideal victims. Two cases illustrate this point. In 2018, the Spanish influencer Dulceida faced a barrage of negative messages for posting photos in a jacuzzi in Africa during a drought (El País, 2018b). In the same year, another major Spanish influencer, Estefi Unzu, known as "*Verdelis*," was accused of exploiting her underage children on her YouTube channel. Detractors blamed her for child exploitation and called for the closure of her channel (ABC, 2018). In both cases, the harassers received public support because they were perceived as avengers of the influencers’ disreputable behaviour. Moreover, the responsibility for the harassment is diffused among a large group of unidentified offenders. Such an avenging and faceless group does not create an ideal victim, as Christie (1986) argued. Finally, the followers of influencers could perceive that the influencers themselves are to blame for the harassment they face. As influencers usually share information about themselves with their followers, they could be stripped of their ideal victim status if the victimisation is perceived as a result of freely chosen activity and exposure, rendering them as non-ideal victims.

## Study 1: Self-Reported Victimization among Influencers

The aim of this research is to investigate the victimisation of Instagram influencers in Spain. Through an online survey, this initial study analyses the incidence of victimisation among influencers in the past year, its impact on their psychological well-being and professional activity, as well as influencers’ responses to harassment. The study focuses on the Instagram platform, which was chosen for three reasons. Firstly, influencers’ posts on Instagram are characterised by greater spontaneity and everydayness than other social media sites like Twitter or YouTube. Influencers use the platform’s story feature to share mundane moments from their daily lives, revealing intimate details such as their homes, social relationships, and daily routines. Secondly, the influencer constructs their image on Instagram using multiple message formats, including photographs, short videos, reels, and texts, which amplifies users’ exposure to the influencer’s content. Thirdly, unlike other platforms like Twitter, Instagram allows followers to send private direct messages to influencers, which may encourage potential harassers to send hate messages.

### Method

#### Data

Study 1 utilises data gathered from a survey of 76 Spanish influencers. Eligible participants were influencers whose primary economic activity was in Spain. Data collection took place through an online questionnaire hosted on *SurveyMonkey*. We adopted a non-probability purposive sampling approach, combined with snowball sampling. Firstly, we created a list of potential participants for the study based on the number of followers and type of profile, aiming to encompass *nano influencers* (5,000 to 10,000 followers) through to *mega influencers* (over 2.5 million followers). The list comprised 150 Instagram influencers. To contact participants, we created a bespoke Instagram account, ‘*@acosoinfluencer*’ (refer to Appendix 1). We then sent invitations to participate in the study, along with a link to the questionnaire, through this account (see Appendix 2). Furthermore, we requested that participants share the questionnaire with their fellow influencers. As we had a low response rate, we contacted fourteen Spanish influencer agencies to assist us in disseminating the questionnaire. Only one agency responded, agreeing to share the questionnaire with their cohort of influencers. The questionnaire was open from October 2021 until July 2022.

We received a total of 98 responses to the online questionnaire. However, we had to exclude 22 incomplete responses, resulting in a final sample of 76 influencers who answered all the questions in the questionnaire. While this sample size is small, it provides an exploratory value as the first criminological study to gather data on reported victimisation among Instagram influencers. Our sample had a higher proportion of female participants (68.4%) than the population of influencers in Spain (56%) (Influencity, 2019). We attempted to address this limitation by contacting male influencer accounts but were unsuccessful. The age of our participants ranged from 14 to 56 years (Min. = 14, Q1 = 24, Q2 = 27.5, M = 29.2, SD = 7.6, Q3 = 34, Max. = 56). Based on their educational level, participants held secondary school (3.9%), high school (21.1%), vocational training (9.2%), Bachelor’s degree (36.8%), Master’s degree (26.3%), and PhD (2.6%). The majority of participants identified as heterosexual (81.6%), while 10.5% identified as bisexual and 7.9% as homosexual.

**Fig. 1:**
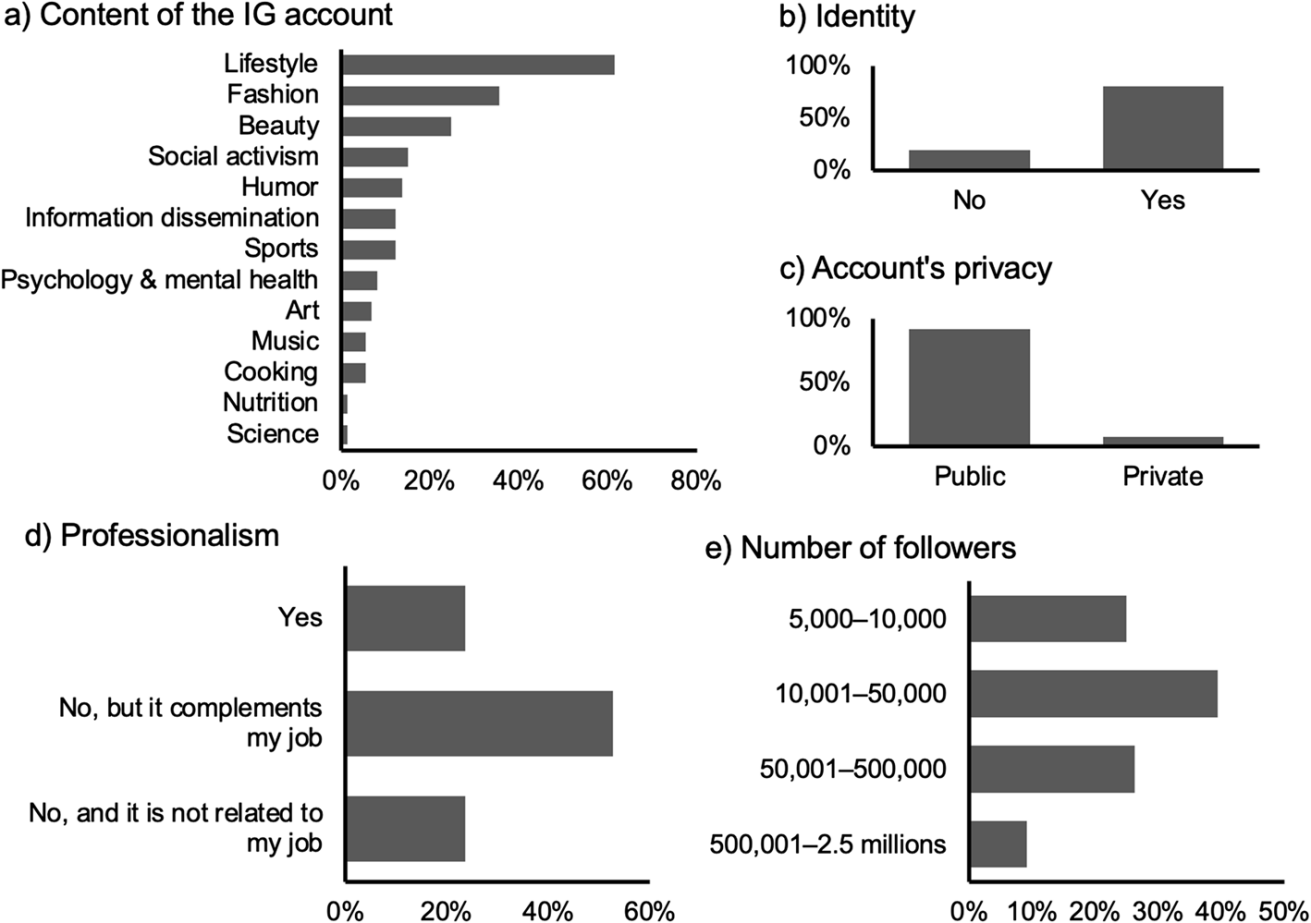
Profile of influencers participating in the study according to **a**) content they post on their Instagram accounts, **b**) whether their account reveals their identity, **c**) privacy settings of their accounts, **d**) professionalism, and **e**) the number of followers

In terms of their Instagram accounts, 25% of the influencers surveyed were classified as nano influencers (5,000–10,000 followers), 39.5% as micro influencers (10,001–50,000 followers), and 26.3% as meso influencers (50,001–500,000 followers), while only seven participants reported having between 500 k and 2.5 million followers, making them macro influencers. No mega influencers (with over 2.5 million followers) were included in our sample. A vast majority of the influencers (92.1%) had public accounts, and 80.3% revealed their true identity on Instagram. Most of the influencers described their accounts as focusing on lifestyle (61.6%), fashion (35.6%), and beauty (24.7%). Additionally, a considerable proportion of the influencers mentioned social activism (15.1%), humour (13.7%), information dissemination (12.3%), sports (12.3%), psychology and mental health (8.2%), art (6.8%), music (5.5%), cooking (5.5%), nutrition (1.4%), and science (1.4%). The participants’ profile can be seen in Fig. 1.

#### Questionnaire

The current study aims to investigate the victimization experienced by influencers, as well as the effects and responses to online harassment on Instagram. Specifically, the survey questionnaire focused on ten different types of harassment based on cyberbullying victimization research conducted in Spain (Zych et al., 2016), such as insults, threats, hacking, and stalking behaviours. Respondents were asked to indicate the frequency of these events over the past year using a five-point Likert scale ranging from "never" (0) to "every day" (4).

Second, we assessed the psychological and professional impacts of online harassment on influencers. The psychological impact was evaluated using 20 items adapted from the Impact of Event Scale-Revised (IES-R, Weiss & Marmar, 1997). These measures were adjusted to reflect the context of this study (i.e., online harassment) and to prompt participants to respond to the questions. The response options were limited to yes (1) and no (0). The list of 20 items demonstrated good internal consistency (α = 0.79). We also measured the professional impact of online harassment by asking the participants, "Due to the harassment, in the past year, did you suffer economic losses?", "Did your business get negatively affected?", and "Did you have to give up collaborations with brands or start a new project?". The response options were yes (1) and no (0).

The third section of the study involved measuring the victim’s responses to online harassment. Specifically, participants were asked "Which of the following behaviours do you perform in response to harassment by a follower?" and presented with a list of 11 possible responses. An additional "other" option was also provided, with three influencers indicating that they had "blocked the account of the harasser." Additionally, we inquired as to whom the participants shared their experiences of victimization, and offered a list of 12 different categories of acquaintances for them to choose from.

In order to enhance the comprehension of the participating influencers’ profile, we inquired about the content featured in their Instagram accounts, whether their profiles disclosed their identities, their number of followers, the extent of their privacy settings, and employment status. The detailed information is illustrated in Fig. 1. Additionally, socio-demographic data were gathered regarding the influencers’ gender, educational level, age, and sexual orientation.

#### Ethical and Methodological Challenges

The present study faced several challenges stemming from two main factors. Firstly, the study of online victimization is a sensitive topic (Díaz Fernández, 2019) that poses ethical and methodological challenges. Online victimization encompasses transgressive and harmful behaviours, which participants may not wish to recall. Secondly, the study focuses on social media influencers, a population that seldom provides self-reported data (Wróblewski & Grzesiak, 2020 being an exception). The scarcity of their participation in scientific studies may be due to academics’ limited access to them. Additionally, as a population that is highly exposed to public opinion, we expected them to exercise greater caution. Therefore, we assured participants that the survey was entirely anonymous to instill confidence in our research. We did not collect personal data, and responses were presented in a statistical format. However, despite our efforts to convey our ethical standards, the response rate was low.

### Results

#### Victimization for Online Harassment

**Fig. 2:**
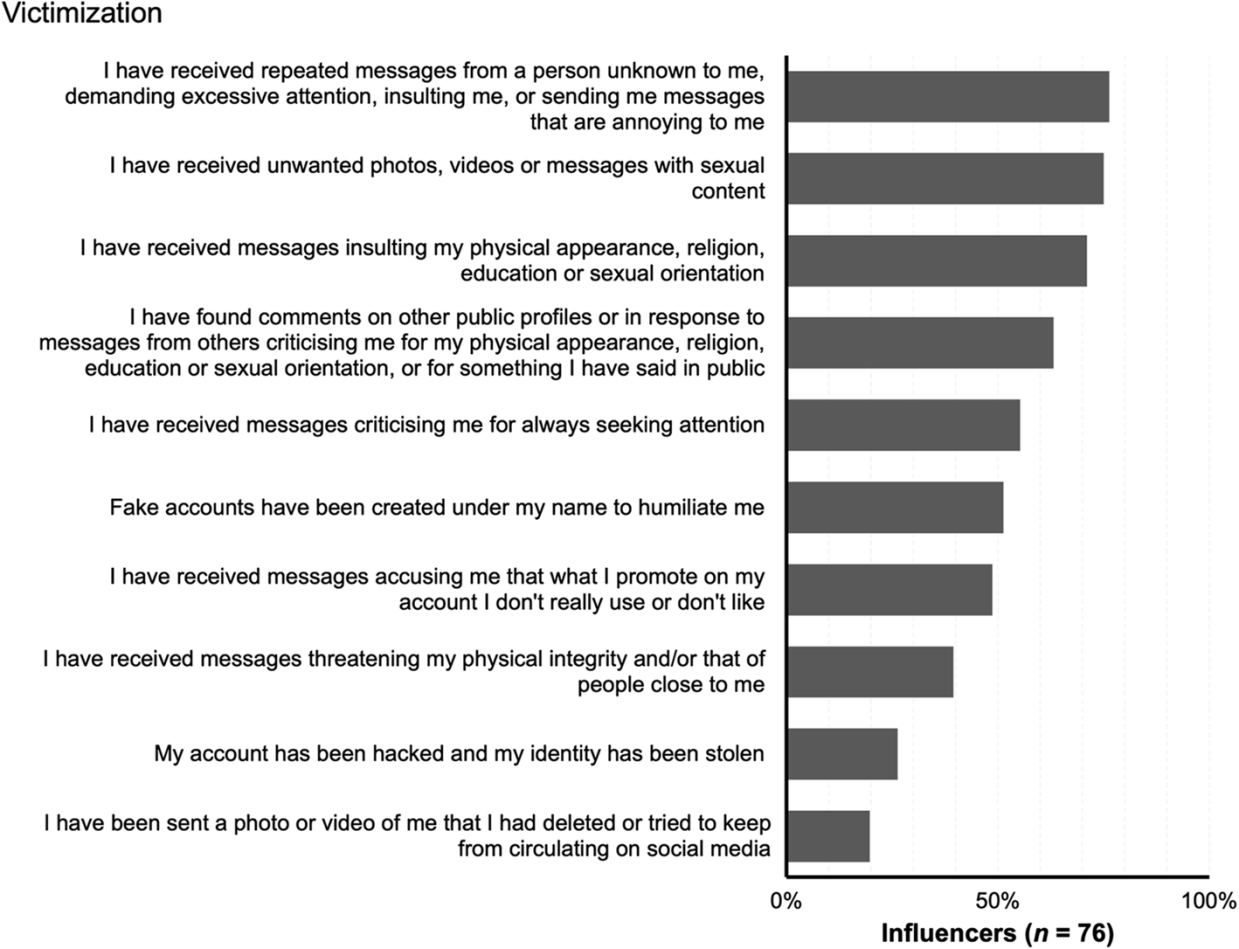
Percentage of influencers who have suffered each type of online victimization

The results of online harassment victimization are presented descriptively in Fig. 2. Each type of harassment was dichotomized into absence or presence, with ‘never’ recoded as ‘absence’ and any frequency reported as ‘presence’. The most common form of online harassment experienced by influencers is repeated messages from an unknown individual who demands attention, sends insulting messages, or is otherwise annoying (76.3%). This type of online harassment can be classified as stalking. The next two most frequent forms of harassment are unwanted pornography, which can be characterized as sexual harassment (75%), and insults (71.1%). In contrast, the least common forms of victimization are related to privacy violations, such as the diffusion of private material and hacking. Men reported the highest proportion of online harassment (32.9%), followed closely by women (31.6%). Only six influencers (7.9%) reported not experiencing any form of online victimization.

Demographic variables were analysed for differences in online harassment. In addition to testing for differences in each type of harassment, a new variable was computed with the sum of all types of harassment (Min. = 0; M = 5.3; Mode = 7; Max; = 10; SD = 2.6). No significant differences were found in the influencers’ gender, except for the receipt of photos or videos in which the influencer appears and that they tried to keep from circulating on social media^5^: 33.3% of male influencers versus 13.5% of female influencers [*X*^2^(1, *n* = 76) = 4.09; *p* = 0.043]. The mean number of victimisations suffered did not differ significantly according to gender (p = 0.73); both male and female influencers suffered an average of five types of victimisations (*M*_male_ = 5.4; *M*_female_ = 5.2). Regarding educational level, a statistically significant association was found only between the receipt of photos or videos that the influencer tried to keep from circulating on social media and lower levels of education among influencers [*X*^2^(5, *n* = 76) = 13.85; *p* = 0.017]. None of the influencers with PhD or Master’s degrees reported being victimised in this way. Furthermore, 82.1% of influencers who received unwanted pornography identified as ‘heterosexual’. However, when examining the proportion of victimised influencers within specific sexual orientations to control for sample size, this result differed: 87.5% of bisexual influencers reported having received unwanted pornography, compared to 51.6% of heterosexual and none of homosexual influencers. The ANOVA test also revealed statistically significant differences in the mean number of online harassment types suffered by influencers according to their sexual orientation [*F*(2, 73) = 4.83, *p* = 0.011, η^2^ = 0.12]. Bisexual influencers reported a larger number of online victimisations (M = 7.1, SD = 1.9) than heterosexual (M = 5.2, SD = 2.5) and homosexual (M = 3, SD = 2.3) influencers.

It was found that lifestyle influencers who posted content on their accounts received a greater number of messages accusing them of promoting products that they did not actually use, which is referred to as accusations of untrustworthy advertising [*Φ* = 0.38, *p* = 0.001]. This was the case for 29 out of 45 lifestyle influencers (64.4%), as opposed to 8 out of 31 non-lifestyle influencers (25.8%). No significant differences were found with regard to the content of the influencers’ Instagram accounts, their real identity disclosure, or the privacy of their accounts. However, a relationship was observed between the degree of professionalism of the influencer and certain types of online harassment. For instance, 65% of influencers who used Instagram to complement their job reported being victimized by followers creating fake accounts, compared to 38.8% of full-time influencers and 33% of non-professional influencers [*X*^2^(2, *n* = 76) = 6.44; *p* = 0.040, *V* = 0.29]. Full-time and part-time influencers were found to be more likely to receive messages with insults (83.3% and 80%, respectively) than non-professional influencers (38.3%) [*X*^2^(2, *n* = 76) = 11.93; *p* = 0.001, *V* = 0.39]. The majority of full-time influencers (66%) received accusations of untrustworthy advertising, compared to 55% of part-time influencers and 16.6% of non-professional influencers [*X*^2^(2, *n* = 76) = 10.35, *p* = 0.006, *V* = 0.37]. In addition, statistically significant differences were found in the mean number of victimizations per professionalism level [*F*(2, 73) = 6.73, *p* = 0.002, η^2^ = 0.16], with part-time influencers reporting the highest number of types of online harassment (M = 6, SD = 2.4), followed by full-time (M = 5.4, SD = 2.8) and non-professional (M = 3.5, SD = 1.9) influencers.

**Fig. 3:**
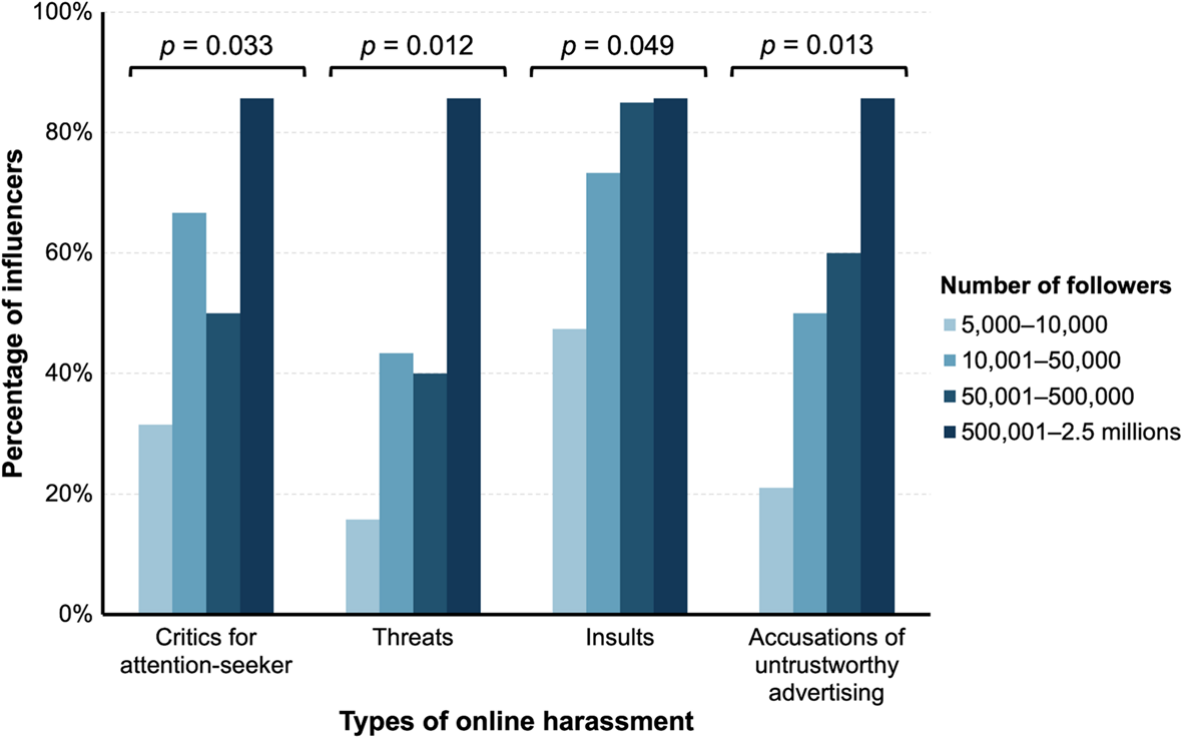
Victimization among social media influencers per types of harassment and number of followers

In this study, we investigated whether there was an association between the level of public exposure of influencers, measured by their number of followers and the amount of personal information they share on their Instagram account, and online harassment. We found significant differences in the proportion of influencers who received harmful messages based on their number of followers. Specifically, those with a larger number of followers reported receiving higher levels of harmful messages related to attention-seeking [*X*^2^(3, *n* = 76) = 8.74; *p* = 0.033, *V* = 0.34], threats [*X*^2^(3, *n* = 76) = 10.92; *p* = 0.012, *V* = 0.38], insults [*X*^2^(3, *n* = 76) = 7.88; *p* = 0,049, *V* = 0.32], and accusations of untrustworthy advertising [*X*^2^(3, *n* = 76) = 10.69; *p* = 0.013, *V* = 0.38]. Additionally, the ANOVA test revealed that influencers with 500,000 to 2.5 million followers reported a higher number of online harassment behaviours compared to those with lower numbers of followers (see Fig. 3). Furthermore, we found a positive correlation between the amount of personal information shared by the influencer on their Instagram account and the number of victimizations they experienced, *r*(74) = 0.29, *p* = 0.01. The influencers who shared more personal information were more likely to report being victimized (Min. = 0, Mode = 5, *M* = 5.8, *SD* = 3.8, Max. = 16).

To explore potential factors related to the frequency of victimization experienced by influencers, we conducted a negative binomial regression analysis, as this model is recommended for count variables (Osgood, 2000). Specifically, the dependent variable in our study is the number of instances of harassment that each influencer received. We included four explanatory variables in the model: exposure to the public, total content shared by the influencer, age, and sexual orientation based on the variables that might be associated with victimization according to the RAT. Initially, we attempted to fit a Poisson regression model, but the dependent variable did not meet the equidispersion assumption required for this type of model (Hilbe, 2017). Thus, we chose a negative binomial regression model (NB2) to correct for overdispersion in the data.

We utilised an NB2 regression model with a logarithmic link function and a deviation-based scaling parameter method. To estimate the results, we used the robust estimator to obtain the covariance matrix and employed the likelihood ratio method to calculate the chi-square statistic, which are commonly used parameters in such analyses (Aeberhard et al., 2014; Maldonado-Guzmán, 2022). Following the model run, the Omnibus test indicated that the model with predictor variables significantly outperformed the null model (Chi-square likelihood ratio: 14.84, p < 0.05).

The findings from the NB2 regression model revealed significant relationships between the number of harassment victimisations and exposure to the public, the quantity of shared content, and the influencer’s age. Exposure and content were positively associated with the number of victimisations, while age showed an inverse correlation. Specifically, younger influencers experienced a higher frequency of harassment incidents. The model results are displayed in Table 1. It should be noted that the regression coefficients’ exponential values must be calculated due to the logarithmic link function used.

**Table 1:** Results of the negative binomial regression for number of harassment victimizations

Variable	Coefficient	*p*-value	95% CI	
			L-CI	H-CI
Intercept	3.643	0.008	.568	6.71
Exposure to public	0.77	0.042	.006	.170
All shared contents	0.747	0.026	.138	1.401
Age	- 0.072	0.009	.219	1.018
Sexual orientation	.942	0.375	-.757	3.144

Table 1 displays the results indicating that a one standard deviation increase in exposure to the public corresponds to a rise of 1.08 harassment victimisations (exp(0.077) = 1.08), and a one standard deviation increase in content sharing leads to an increase of 2.16 victimisations (exp(0.747) = 2.16). In addition, an increment of one standard deviation in the influencer’s age results in a reduction of 1.07 harassing messages received. The analysis demonstrated that the sexual orientation of the influencer did not significantly contribute to the number of victimisations experienced.

#### Impact of Online Harassment

Among the cohort of influencers who reported experiencing any of the psychological consequences listed in the survey, 49.3% endeavored to repress the events. Despite their attempts to disassociate from the harassing behavior, those who reported suffering psychological ramifications described feeling irritable and angry (33.3%), melancholic (29%), vigilant (24.6%), preoccupied with the event (23.2%), and anxious (20.3%). The repercussions of harassment also extended to the influencers’ professional pursuits. Of those who indicated that their career was affected, 89.1% abstained from collaborating with brands or initiating novel projects to avoid being harassed. In addition, 19.6% experienced financial losses as a consequence of the harassment.

Significant statistical differences were observed between gender and the psychological impact on victims in our study. Only women reported experiencing a sense of feeling blocked, manifesting as a state of shock, after a harassment incident [*X*^*2*^(1, *n* = 70) = 4.71, *p* = 0.003]. Conversely, men were more inclined to feel that the incident had not occurred or was not real [*X*^*2*^(1, n = 70) = 4.76, *p* = 0.029]. Gender disparities were also detected in the economic impact of harassment, as 19.6% of women reported incurring financial losses, whereas men did not report such losses [*X*^*2*^(1, n = 70) = 5.39, *p* = 0.02].

In relation to the profile of the influencer, our study identified differences solely in the psychological impact experienced. Those with a larger following, ranging from 50,001 to 2.5 million, were more prone to avoiding discussions about the harassment they encountered than those with fewer followers [*X*^*2*^(3, n = 70) = 15.04, *p* = 0.002]. Moreover, individuals who identified as professionally engaged in producing online content frequently reported struggling with sleep disturbances as a result of online harassment [*X*^*2*^(1, n = 70) = 9.73, *p* = 0.008].

#### Reactions to Online Harassment

The victims’ reactions were analysed with respect to two variables: (i) personal measures or actions taken, and (ii) the individual with whom they shared their experience of harassment. The most commonly reported personal measures taken by victims were: ignoring the harassing behaviour (74.2%), confronting the harasser (37.9%), reducing the amount of personal information shared online (31.8%), and minimizing or denying the problem (31.8%). Communication about the harassment experience generally occurred within the victims’ closest social circles, with 69.1% of victims sharing their experience with a friend, 54.4% with a partner, 48.5% with a family member, and 26.5% with another influencer.

Statistically significant gender differences were observed among the victims. Female influencers are more likely to gather evidence of harassment (89.5%), for instance by taking screenshots, compared to male influencers (10.5%) [*X*^2^(1, n = 70) = 6.53; *p* = 0.011]. Victim influencers who work full time are the ones who usually confront harassers by responding to their messages (44%) [*X*^2^(2, n = 70) = 7.64, *p* = 0.022] or retaliate against them by exposing their comments and identity on their own accounts to publicly denounce the harassment (58.3%) [*X*^2^(2, n = 70) = 7.99, *p* = 0.018]. Influencers with the most followers, ranging between 500,001 and 2.5 million, commonly share their harassment experience with their legal representatives [*X*^2^(3, n = 70) = 8.19, *p* = 0.042]. When participants identify as professional social media users and have been subjected to harassment, more than half (62.5%) sought the assistance of a therapist [*X*^2^(2, n = 70) = 7.19, *p* = 0.027].

## Study 2: Online Ethnography of Influencers’ Instagram Accounts

### Method

In the second study, we conducted a virtual ethnography through observation and thematic content review.

#### Sample

The study comprises 260 social media influencers’ accounts in which the construct of ‘influence’ was operationalised by their Instagram followers. To be eligible for inclusion, the influencer’s account was required to have a minimum of five thousand followers. The range of followers in the sample varied between 6760 and 358 million. The non-probabilistic multistage sampling method was utilised to collect the sample in three stages. Firstly, quota sampling was used to ensure the representation of different thematic profiles, such as sports, fashion, and science. Secondly, snowball sampling was employed to identify additional profiles by extracting them from the accounts of previously selected influencers and the social network’s suggestions. Finally, convenience sampling was used to select certain influencers based on their online behaviour and previous public reports of online harassment.

#### Data Collection

The data for this study was gathered from two primary sources. Firstly, Instagram was selected as the most commonly used social network by influencers, as reported by Casaló et al. (2020). Secondly, public discussion forums hosted on various websites were analysed to examine comments and enquiries related to celebrities. Data collection from the first source involved gathering both regular profile posts and twenty-four-hour-long stories within the period spanning from April 2, 2021, to May 7 of the same year. A total of 2,050 images and/or videos were collected, which included both regular posts and stories.

In relation to the discussion forums, information on 90 influencers from the sample was identified in a total of 76 forums. A non-participant approach was used to examine the forum posts produced during the month of April 2021, resulting in the collection of 867 images. The combined data obtained from the two sources resulted in a total of 2,917 images and videos, which comprised 1,921 images capturing responses to Instagram posts and a total of 4,498 associated comments. Furthermore, 129 images and videos were collected from Instagram stories, while 867 images featured photographs that contained 966 distinct messages.

#### Data Analysis

The objective of this study is to examine the harassment experienced by influencers by analysing comments made by followers or anonymous individuals on their social media accounts, as well as in public forums. A manual analysis by topic has been conducted, with a focus on six main thematic areas: (i) harassment; (ii) precipitating factors; (iii) justification of abuse; (iv) intervention of witnesses; (v) response of the victim; and (vi) effects of the attack. To protect the privacy of the selected profiles, the comments were coded using a unique number assigned to each collected image or video. Depending on the data source, a letter was assigned, with ‘P’ representing posts, ‘S’ representing stories, and ‘F’ representing forums. Personal names have been anonymised using the letter X.

#### Ethical Considerations

The present study encompasses various ethical considerations that must be addressed. Past literature has contended that individuals who participate in online activities on public domain websites should not have an expectation of privacy (Watson et al., 2007). In this regard, the current research analysed messages published on a public forum that does not require login credentials to access, similar to previous studies (McLean & Griffiths, 2019). Regarding the information obtained from Instagram, in line with previous virtual ethnographies carried out on this social network (Sidoti, 2021), all accounts examined were identified as public profiles. Moreover, it is understood that this publicity extends to all accounts that interact with the influencer’s account, as their comments are made on a public profile.

Efforts were made to ensure that the research was not conducted as a covert virtual ethnography. At the outset of the data collection, a public profile on Instagram was established to inform about the research’s existence, aims, and data collection methods. Moreover, we sent a direct message to all observed influencers to personally explain the research. Only four of them responded, granting explicit consent to participate in the study.

Furthermore, during April, 18 publications were shared on our profile concerning online harassment and outlining the study’s characteristics (see Appendix 1). Influencers were tagged in these publications to inform them about the research, resulting in seven additional influencers providing express consent. Throughout the study, we respected Instagram’s terms and conditions of use. All identifying information, including names, were anonymised to protect the participants’ identities.

## Results

The principal forms of harassment observed in this study are expressed through derogatory, humiliating, and offensive comments targeting the behaviour and attitudes of the social media influencers. The criticism is frequently aimed at the victim’s personal life, such as their parenting style, clothing choices, or physical appearance. For instance, an example comment from a forum participant reads: "She is useless and a bad mother" (F334). Notably, female influencers are frequently the focus of comments intended to belittle their accomplishments, often by justifying them solely based on their relationship with a man. As an illustration:

- P526: You only rose to affluence thanks to [*name of male celebrity*], and now you live among the rich. Before [*name of male celebrity*], no one even knew you existed.
- F1010: Hahaha, if you didn’t have a wealthy boyfriend, you’d be eating cold pizza for breakfast.
- F820: Wife of. You are nothing but.

In the most severe instances, we have come across comments that instigate suicide and even target family members and/or acquaintances. For instance, one comment on a forum read: ‘You’re truly repugnant [*name of the influencer*] […] You resemble a desperate elderly man with your pudgy little body and unappealing clothing choices […] Your dirty old man face is a turn-off’ (F317). Another comment on a post read, ‘Shoot yourself bro [*brother*]’ (P438), while a further one read, ‘THE MONSTER’S DAUGHTER WHO ONLY CARES ABOUT MONEY… IS DISGUSTING!’ (P217).^6^

Haters provide justifications for their attacks on influencers, usually based on two primary reasons. Firstly, they argue that the victims’ personal and private information is publicly and voluntarily exposed. For instance, as one follower questioned, "If it is such a personal matter for us to criticise, why does [*the influencer*] expose it?" (F562). Another follower added, "Their values are based on making a living from showing their life, ah! and, therefore, bearing with what the public says" (F522). When accused of jealousy, they deny it and respond with comments such as, "Envy, the easy recourse. Not envy, criticism in public social networks. If they don’t like it, they should set an example" (P1921). Secondly, haters often excuse themselves by attributing blame to other haters or downplaying the importance of their attacks. For example, "Criticising is one thing, threatening and harassing is another. What we do here is talk like in any group of friends" (F191).

The aforementioned justifications are frequently accompanied by precipitating factors. Our research has identified two primary factors, which include feelings of disgust, anger or jealousy toward the luxurious lifestyle that the influencers enjoy or negative attitudes or behaviours that are viewed as poor examples for society, such as violations of traffic regulations or non-compliance with measures related to the SARS-CoV-2 virus. These factors can be seen as facilitators of harassing behaviour, as demonstrated by the following examples:

- F318: It really pisses me off how these people live, doing nothing. (jealousy)
- F302: (…) I just wish they’d run out of money and have to struggle like the rest of us. (jealousy)
- P1308: Using a baby to get likes is a crappy thing to do. (judgemental comment on behaviour)
- F473: These idiots act like there are no restrictions or pandemic, as usual. (angry at the lack of equity)

In response to receiving these harassing messages, influencers may react in various ways. Some influencers choose to directly respond to the messages they receive. For instance, a popular female influencer received a critical comment about her legs ("you have a nice body but the legs are horrible", P1177) to which she replied, "These comments do not contribute anything positive. I will tell you that I love my legs mainly because they allow me to walk." At times, influencers publish posts specifically addressing and condemning online harassment or create stories where they expose the situation and share their opinions on the matter. As a result of experiencing harassment, influencers often express feelings of concern for their loved ones’ well-being and may decide to reduce the amount of content they post on social media. One influencer explained, "The moment [*harassment*] can affect someone around me…the feeling of guilt, or a little bit of protection, has affected me at times" (S98). Another influencer admitted to self-censoring their Instagram content due to harassment, saying "I suppose that because of these things [*harassment*], I tell less and less about [*name of the partner*], about my private life, and maybe I felt more distant in that sense." In one case, we observed one example of a celebrity seeking support from their followers to combat online harassment:

- P1696: Let’s speak out to put a stop to the online abuse and harassment. No one should have to put up with being treated badly or being called names. It’s becoming more and more common to see and experience this kind of behaviour on social media, and it seems like no one is doing anything to stop it. We need to speak out against these hostile attitudes and demand that the social media companies take urgent action.

## Discussion and Conclusions

Despite the extensive body of literature on online harassment against anonymous individuals (e.g., Choi et al., 2022; Lwin et al., 2012; Moneva et al., 2020; Näsi et al., 2017) and more high-profile groups such as journalists (e.g., Chen et al., 2020; Holton et al., 2021; Miller & Lewis, 2022), as well as the growing academic interest in attacks against influencers (e.g., Duffy et al., 2022; Martínez Valeiro & Mayagoitia Soria, 2021), the present study is, to the best of our knowledge, the first to provide an empirical criminological analysis of online harassment against Instagram influencers. This article reports on two mixed-methods exploratory studies that provide information on the types of victimization, the profile of aggressors, the profile of influencer victims, the justifications for abuse, and its effects. One study analysed victimization as reported by the victims themselves, using a victimization survey, while the other examined the various forms of harassment behaviour as manifested in the toxic communication between users and influencers. The aim is to provide an exploratory understanding of victimization from the perspective of those who experience it, as compared to the comments of haters on Instagram. However, a key limitation is the inability to draw a direct association between the responses of the surveyed influencers and the hate messages collected through virtual ethnography, as these represent different individuals.

According to the findings of both studies, it was discovered that influencers are frequently subjected to online harassment from their followers. The primary form of harassment directed at influencers involves the use of insults and derogatory, humiliating, and repeated comments, which are often experienced on a daily or weekly basis. These results are consistent with those obtained by Lewis et al. (2020), who discovered that the most common forms of harassment against journalists are offensive name-calling (12.8%) and attempts to embarrass them (10.8%). Our results are also consistent with other studies involving people without social influence or power. For instance, verbal abuse and humiliation are the most common types of online harassment directed at college students (Finn, 2004), as well as Black and Latin women (Francisco & Felmlee, 2022).

The findings are consistent with prior research indicating that having a prominent online presence increases the risk of online harassment for certain professions, such as politicians (Farrell et al., 2020; Southern & Harmer, 2021), academics (Celuch et al., 2022), and journalists (Chen et al., 2020; Miller & Lewis, 2022). Our results also support the RAT (Cohen & Felson, 1979), as we found that professional content creators and highly-followed influencers are more vulnerable to online harassment. The primary risk factor for full-time influencers is the number of followers and their business model. Since their income is generated from their social media activity, they may be less likely to take protective measures against online harassment, although some exceptions exist.^7^ Nonetheless, future studies should aim to increase the sample size to achieve greater representativeness of the total influencer population.

This study concludes that online harassment has a detrimental impact on the lives of influencers. The psychological effects reported by the influencers align with previous studies on online harassment, including anxiety (Tynes & Giang, 2009), stress (Staude-Müller et al., 2012), and depression (Feinstein et al., 2014). Furthermore, the study found that over 50% of the influencers surveyed had refrained from collaborating with brands or started new projects to escape harassment. This behaviour has been linked in the literature to efforts to avoid harassment and protect their reputation by turning down new career opportunities (Adams, 2018).

The findings of both the self-reported victimization study and the online ethnography suggest that influencers are often viewed as non-ideal victims. Our investigation revealed that influencers evoke both envy and contempt, which are utilized to rationalize derogatory and harassing behaviour. Harassers often express low warmth and low competence sentiments towards influencers (Fiske et al., 2002). The feeling of contempt towards the influencer figure contrasts with their perception of their high standard of living. Harassers express a dislike for influencers’ lifestyles and view them as unjust. They believe that influencers have not undergone enough personal training or exerted sufficient effort to experience such standards of living. The perception of low competence stems from the idea that influencers have obtained their wealth by ‘selling out their life’. These claims may be linked to inequality aversion (Fehr & Schmidt, 1999), as followers and society may feel that the negative criticism influencers receive counterbalances the excess of their wealth and living standards compared to the general population. Nonetheless, further research is needed to examine this hypothesis more closely.

Both studies reveal that influencers do not seem to fulfil the ideal victim criteria for engaging in a reputable project. The evidence shows that influencers face criticism for what they promote on their social media and their personal choices, such as their parenting style. This finding is consistent with previous research on anti-fan communities (Duffy et al., 2022). By removing influencers from the ideal cyber-victims list, this result may be linked to the notion of fair punishment (Dagger, 1993). When an influencer’s behaviour is regarded as unethical and violates social norms, and it is publicized online, vexatious comments and harassment may be viewed as justifiable. Other studies also support this idea. For instance, a study on the leakage of celebrities’ sexual images found that some people regret that privacy violations only matter when people are famous because "the government, the rich, and the famous get away with worse all the time" (Marwick, 2017, p. 188).

Finally, online harassment against influencers typically lacks a single identifiable offender. Instead, it is an anonymous collective of numerous harassers that contributes to the overall amount of harassment that the influencer receives. This can lead to a diffusion of responsibility and moral disengagement among the harassers (Bandura, 1999), which makes it difficult to categorize influencers as victims. This lack of social recognition as legitimate victims can result in negative consequences, such as secondary victimization dynamics (Tamarit Sumalla, 2013), a lack of social support, and blame (Van Dijk, 2009). These processes can amplify the negative impact of victimization by depriving influencers of emotional and material support, such as compensation schemes (Strobl, 2004). Furthermore, since the public does not identify these behaviours as harassment, it is possible that prevention and awareness-raising programmes may have less impact on these forms of harassment.

Based on the ideas we presented above, we contend that the non-ideal victim conceptual framework is suitable for comprehending why harassment directed towards a particular group –i.e., influencers– might be more socially accepted while harassment targeting anonymous individuals is more condemned. The ideal or non-ideal status of the victims may influence how they are categorised, which can in turn impact the societal response to their victimisation. Bullying, for example, is a form of harassment that is generally denounced. This may be partially because schoolchildren victims tend to meet all of Christie’s criteria for ideal victims.^8^ As a result, bullying has garnered significant media attention, been the subject of research, and been elevated to a social problem that demands political attention. Nevertheless, in this study, we maintain that exploring other forms of victimisation directed at less popular victims can yield valuable insights into how cyber violence is perpetrated and victimisation processes are managed when victims are not publicly recognised. Furthermore, it remains to be determined to what extent the normalisation of certain levels of violence in interactions with influencers may generate a tolerance effect on social media violence in general via social learning. Therefore, criminology should investigate all forms of antisocial behaviour on social media to provide more comprehensive, evidence-based solutions.

This article is subject to several limitations that warrant acknowledgement. The primary limitation of this research is the small sample size used in Study 1. Influencers, who receive a great deal of social interaction on social media, are notoriously difficult to reach, and as a result, they tend not to respond to survey invitations. Consequently, our results are only an approximation of the extent of online harassment directed at influencers and necessitate more in-depth research. Additionally, our study exclusively focused on self-reported harassment by the victims themselves, as well as on followers’ comments in posts and forums. However, we did not examine the arguments and motivations of the harassers through their own discourse. Future research should therefore sample these subjects to probe their attitudes towards harassment directed at influencers. Doing so would allow for a more rigorous understanding of the harassers and their motivations towards harassment. Lastly, our research indicates that certain behaviours of specific influencers elicit harassment against them; however, the characteristics of these behaviours remain unexplored. Future research should examine this question in greater detail.

This study offers a comprehensive research of the prevalence and nature of online harassment against social media influencers on Instagram. Utilising a combination of self-reported victimisation data and online ethnography, this study characterises influencers as a vulnerable population susceptible to various forms of online harassment. The findings provide empirical evidence supporting the conceptualisation of influencers as non-ideal victims, given their inability to satisfy the criteria necessary to elicit empathy and appreciation from the public. While our results cannot be generalised to the entire population of influencers, they represent a significant advancement in our understanding of online harassment among some of the most publicly exposed individuals in our society. As such, this study serves as a critical contribution to the ongoing conversation about online harassment and underscores the need for further research to fully comprehend the breadth and depth of this societal scourge.

## Data Availability

The datasets generated and analysed during the current study are not publicly available due to the sensitivity of the data and to and to ensure the privacy of the Spanish influencers who have been selected in the qualitative study.

## Appendix 2 Invitation Letter to Participate in the Study Sent to Influencers

Dear [*Name of the Influencer*],

We are a group of researchers from the University of Cadiz and Leiden University who are conducting a study on virtual harassment experienced by individuals with prominent profiles, such as yourself, on the social networking platform, Instagram.

We would like to extend an invitation to participate in our study, which aims to bring attention to the issue of harassment faced by influencers and to propose preventative measures and awareness campaigns to combat this growing problem. The survey should take no more than five minutes to complete. If you have already participated in this study, we extend our heartfelt gratitude for your contribution, and you need not complete the survey again.

Please use the following link to access the survey: [link].

Thank you very much for your participation.

Sincerely,

The Research Team.

## Footnotes

1 Alexandra Pereira is one of the most renowned Spanish influencers in the fashion content creation industry, with a career spanning over a decade. She gained recognition through her lifestyle and fashion blog, which propelled her to international stardom. She currently resides in Dubai and boasts an international profile, collaborating with top brands such as Dolce & Gabbana. With over two million followers on Instagram, she has amassed a significant following on social media.

2 The Vogue Forums was an online platform provided by Vogue magazine for individuals with shared interests in the fashion industry to engage in discussions and exchange ideas. Unfortunately, inadequate moderation of the forum led to an influx of harassment and hate messages directed towards high-profile women such as Alexandra Pereira.

3 “*El hormiguero*” is a Spanish TV program focused on comedy, science, and guest interviews.

4 Elaine Duffy (1995) initially introduced the notion of horizontal violence, which refers to aggressive conduct by individuals from a structurally oppressed group who engage in violence against other members of the same group, such as women. Such individuals may rationalise their violent behaviour based on the personality traits of the victims.

5 Influencers aim to prevent the circulation of specific content, which may occur due to two potential reasons. Firstly, the influencer may have posted the content previously and subsequently removed it. Secondly, the content may include photos or videos taken without their consent in private settings, intended to harm their image.

6 In capital letters in the original in Spanish.

7 Such as the case of the Spanish influencer Marta Pombo (702 k followers in Instagram), who stopped her activity on her personal accounts for months in 2020 due to mental health issues caused by social media (del Río, 2021).

8 The authors acknowledge that there are many more factors that contribute to bullying being a social problem that are not related to the “ideal” status of the victims.
